# Molecular Modeling Studies of the Novel Inhibitors of DNA Methyltransferases SGI-1027 and CBC12: Implications for the Mechanism of Inhibition of DNMTs

**DOI:** 10.1371/journal.pone.0062152

**Published:** 2013-04-25

**Authors:** Jakyung Yoo, Sun Choi, José L. Medina-Franco

**Affiliations:** 1 National Leading Research Lab of Molecular Modeling & Drug Design, College of Pharmacy, Division of Life and Pharmaceutical Sciences, and Global Top5 Research Program, Ewha Womans University, Seoul, Korea; 2 Instituto de Química, Universidad Nacional Autónoma de México, Mexico City, Mexico; Complutense University, Spain

## Abstract

DNA methylation is an epigenetic modification that regulates gene expression by DNA methyltransferases (DNMTs). Inhibition of DNMTs is a promising approach for cancer therapy. Recently, novel classes of the quinolone-based compound, SGI-1027, and RG108-procainamide conjugates, CBC12, have been identified as potent DNMT inhibitors. In this work, we report comprehensive studies using induced-fit docking of SGI-1027 and CBC12 with human DNMT1 and DNMT3A. The docking was performed in the C-terminal MTase catalytic domain, which contains the substrate and cofactor binding sites, in the presence and absence of other domains. Induced-fit docking predicts possible binding modes of the ligands through the appropriate structural changes in the receptor. This work suggests a hypothesis of the inhibitory mechanisms of the new inhibitors which is in agreement with the reported autoinhibitory mechanism. The insights obtained in this work can be used to design DNMT inhibitors with novel scaffolds.

## Introduction

DNA methyltransferases (DNMTs) catalyze the transfer of a methyl group from *S*-adenosyl-L-methionine (SAM or AdoMet) to the carbon-5 position of cytosine residues that result in an epigenetic change [1]. Three active forms of DNMT have been identified in mammals: DNMT1, DNMT3A/3B, and DNMT3L. DNMT1 which is the most abundant of the three is involved in the maintenance of methylation patterns, whereas DNMT3A and DNMT3B are responsible for de novo methylation [2], [3]. DNMT3L is required for the catalytic activity of DNMT3A and DNMT3B, though it lacks catalytic activity because of the absence of conserved catalytic residues [4], [5]. These enzymes regulate gene expression. For example, hypermethylation of the promoter lead to transcriptional silencing of tumor suppressor genes. Therefore, DNMT inhibitors are promising new drugs for the treatment of diseases such as cancer and brain disorders [6], [7].

**Figure 1:**
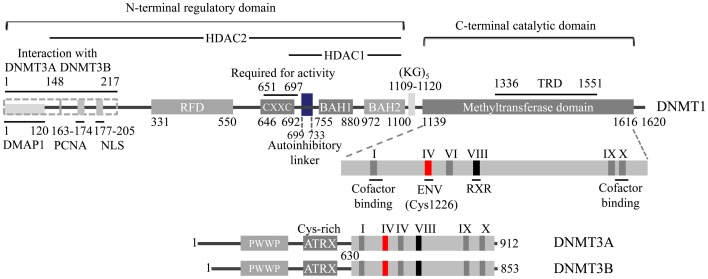
Schematic representation of DNMT1 and 3s. NLS, nuclear localization signal; RFD, replication foci-targeting sequence; BAH, bromo-adjacent homology domain; TRD, target recognition domain; PWWP, a conserved region containing the core tetrapeptide of ‘proline-tryptophan-tryptophan-proline’; ATRX, cys-rich region. Interaction domains of HDAC1, HDAC2, and the DNMT3s are indicated. The methyltransferase domain comprising six most conserved motifs is enlarged.

The structure of mammalian DNMTs with 1616 amino acids can be divided into an N-terminal regulatory domain, and a C-terminal catalytic domain (Figure 1) [8], [9]. The N-terminal domain consist of a replication foci-targeting domain (RFD), a DNA-binding CXXC domain, and a pair of bromo-adjacent homology domains (BAH) (Figure 1) [10], [11]. The C-terminal catalytic domain, which is conserved in eukaryotic and prokaryotic DNMTs, consists of 10 amino acid motifs. The cofactor and substrate binding sites in the C-terminal catalytic domain are comprised of motif I and X and motif IV, VI, and VIII, respectively [12]. The target recognition domain (TRD) which is maintained by motif IX and involved in DNA recognition, is not conserved between the DNMT family (Figure 1).

**Figure 2:**
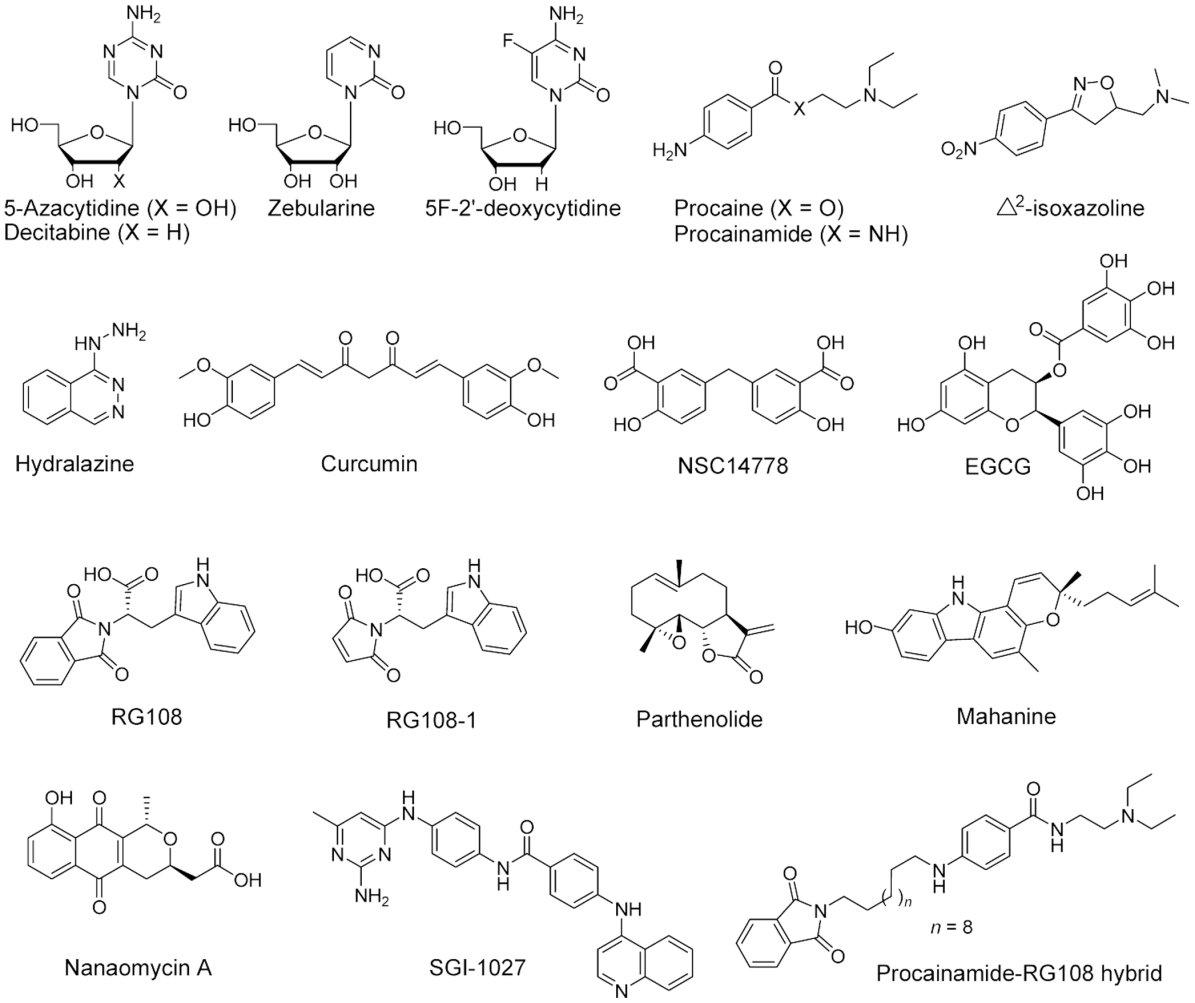
Chemical structures of DNMT inhibitors.

To date, only 5-azacytidine (5-aza-CR, Vidaza®) and 5-aza-2′-deoxycytidine (5-aza-CdR, Dacogen®) are clinically in use for the treatment of certain types of cancer [13], [14], [15], [16]. However, there are still concerns about low specificity and clinical toxicity of nucleoside analogues [16]. To overcome these concerns, it is necessary to discover and develop non-nucleoside DNMT inhibitors. Compounds with different chemical classes are associated with demethylating activity, and some of them were proposed as DNMT inhibitors (Figure 2) [7], [17], [18]. Most of these compounds were identified fortuitously and there are current efforts to search systematically and develop potent and selective compounds [19], [20]. For example, we recently conducted molecular modeling studies to understand the key interactions between the crystallographic structure of the catalytic domain of DNMT1 and known inhibitors [21], [22], [23]. Also, several compounds with new scaffolds were identified from structure-based virtual screening [24], [25], [26].

SGI-1027 is a novel DNA hypomethylating agent with a quinoline-based scaffold (Figure 2) [27]. SGI-1027 directly inhibits DNMT activity competing with the cofactor, SAM. This compound shows comparable inhibitory activity of DNMT1, DNMT3A and DNMT3B (IC_50_ (6–13 µM)) without significant toxicity. However, the molecular modeling study of SGI-1027 is not reported. Only a chemoinformatic-based approach using the similarity profile of SGI-1027 to different chemical databases has been conducted by our group to identify novel scaffolds [28], [29].

New synthetic DNMT inhibitors, based on the conjugation of procainamide to L-RG108 or phthalimide (CBC12 in Figure 2) were reported recently [30]. Among the non-nucleoside analogues, procainamide is a potential DNMT inhibitor approved by the FDA as antiarrhythmic [31], and L-RG108 was identified via virtual screening (Figure 2) [24]. These conjugates had a long scaffold linked by at least six alkyl chains. A docking model of the most potent compound, CBC12, with the crystal structure of DNMT3A/3L was proposed [30].

**Figure 3:**
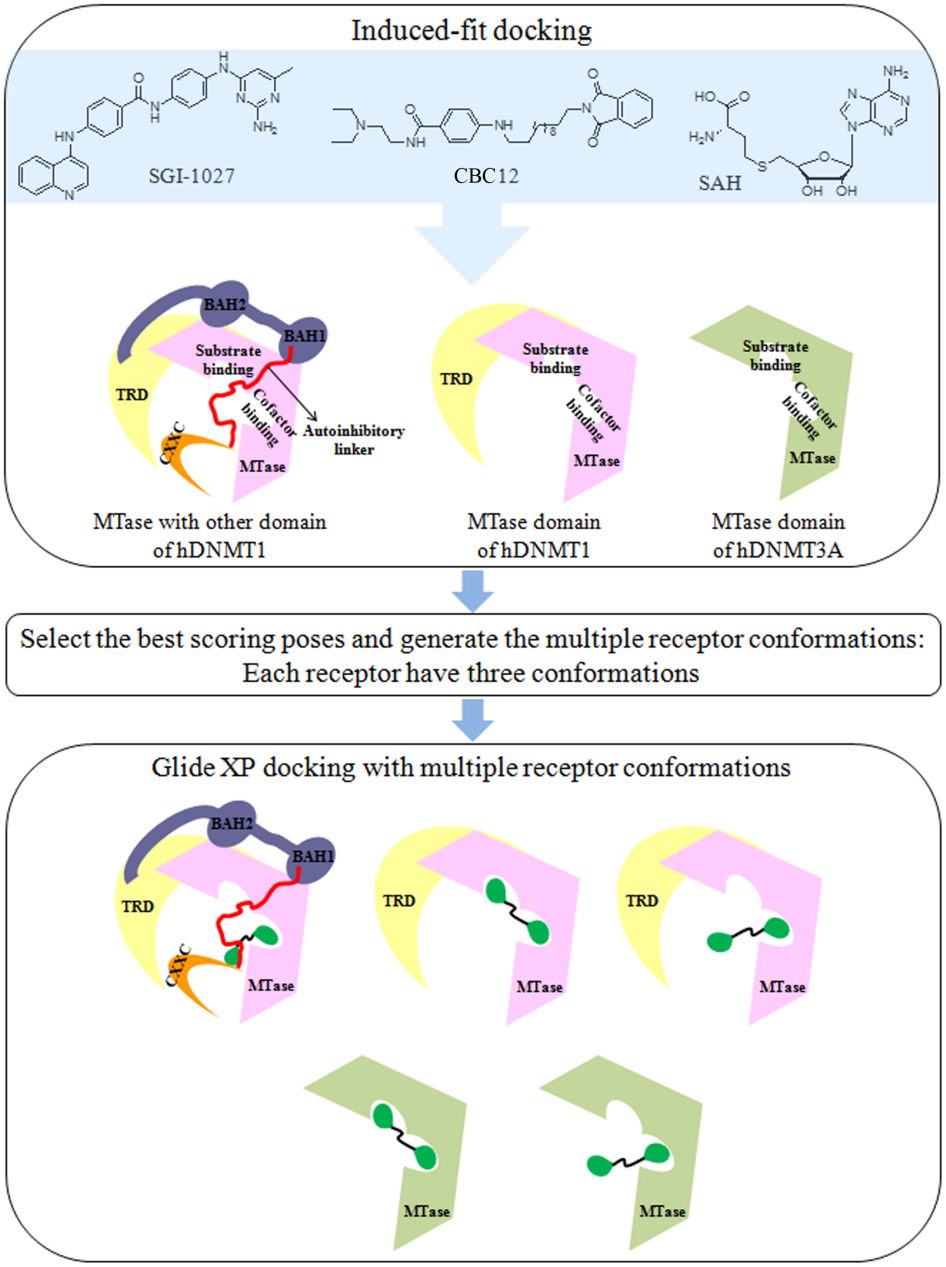
Workflow of the docking study using induced-fit docking and multiple receptor conformations.

Herein, we propose the binding mode of SGI-1027 and CBC12 with DNMT1 and DNMT3A. In order to account for protein flexibility, we employed induced-fit docking (IFD). The crystal structure of human DNMT1 (hDNMT1) with the methyltransferase (MTase) and other domains suggested an auto repressive mechanism according to the positioning of the autoinhibitory linker between unmethylated and hemimethylated CpG dinucleotides (Figure 3).

## Materials and Methods

To predict the docking poses of SGI-1027 and CBC12, we performed induced fit docking (IFD) with DNMT1 and DNMT3A. The MTase domain with and without other domains of DNMT1 were taken into account. The best docking poses of each compound were moved forward for re-docking (Figure 3). The ensemble docking with multiple receptor conformations and regular docking (a single receptor conformation) were also conducted to compare the docking scores and binding modes generated with the different docking methods. *S*-adenosyl-L-homocysteine (SAH or AdoHcy) was used as a reference molecule in each step.

### Preparation of Protein Structures

The crystal structures of hDNMT1 (PDB id: 3SWR) [32] and hDNMT3A-hDNMT3L C-terminal domain complex (PDB id: 2QRV) [4] were chosen to get insights into the different binding modes of SGI-1027 and CBC12 with human DNMT1 and DNMT3A. Of note, the crystal structure of mouse DNMT1 with hemimethylated DNA containing a nucleoside inhibitor is available (PDB id: 4DA4). Despite the fact this structure is in an active form, it was not used in this work because the crystallographic structure does not have the CXXC domain (residues 646–692) and autoinhibitory linker domain (699–733). The full sequence of the crystal structure (4DA4) only has the BAH1, BAH2 and MTase domains (732–1600). In addition, the docking scores of new inhibitors obtained in this work are in agreement with the published *in vitro* data which is for human DNMT1 (see below).

The MTase domain of hDNMT1 was prepared with (sequence 601–1600) and without (sequence 1129–1600) other domains to study the effects of other domains on the interactions of ligands. Protein structures of hDNMT1 and hDNMT3A-hDNMT3L bound to sinefungin (SFG) and SAH, respectively, were prepared using the Protein Preparation Wizard implemented in Maestro (version 9.2, Schrödinger, LLC, New York, NY, 2011) with the following steps [26]: (i) The missing side chains were added to the crystal structure by Schrödinger’s Prime 3.0. [33] (ii) Hydrogen atoms were added and water molecules within 5 Å of the co-crystallized ligand were removed. (iii) Protonation states of entire systems were adjusted to the pH range of 7.0+/−4.0 using Epik. (iv) Hydrogen bond networks and flip orientations/tautomeric states of Gln, Asn, and His residues were optimized with sample water orientations at a neutral state. (v) The geometry optimization was performed to a maximum root mean square deviation (RMSD) of 0.3 Å with the OPLS2005 force field.

### Preparation of Ligands

The chemical structures of SGI-1027 and CBC12 were built using Maestro 9.2. SFG and SAH were extracted from the corresponding crystal structures (PDB id: 3SWR and 2QRV). Ligand structures were submitted to the Polak-Ribiere Conjugate Gradient (PRCG) energy minimization using the OPLS 2005 force field until the energy difference between subsequent structures was 0.001 kJ/mol-Å [34]. The possible tautomers of ligands maintaining original stereochemistry were explored using LigPrep (version 2.5, Schrödinger, LLC, New York, NY). The conformational search of ligands was performed using ‘Fast’ mode implemented in ConfGen (version 2.3, Schrödinger, LLC, New York, NY) with OPLS 2005. The input and output structures were energy minimized. The redundant output conformers (RMSD <1.0 Å) were eliminated.

### Induced-fit Docking (IFD) Procedure

Two hDNMT1-SFG complex structures of MTase domain with (sequence 601–1600) and without (sequence 1129–1600) other domains of 3SWR, and the hDNMT3A-SAH complex structure of 2QRV, were used as starting geometries for the IFD protocol implemented in the Schrödinger software suite [35]. The prepared ligands SGI-1027, CBC12, and SAH were docked into each protein structure using the following steps: (i) The receptor grid was defined as an enclosing box at the centroid of the co-crystallized ligand (i.e., SFG and SAH) to include the cofactor and substrate binding sites. (ii) In the initial Glide docking stage, a soften potential docking with the van der Waals radii scaling of 0.7 for the proteins was performed to retain the maximum number of 20 poses per ligand. (iii) Residues within 5.0 Å of ligand poses were kept free to move in the Prime refinement step, and the side chains were further minimized. (iv) Ligands were re-docked into their corresponding receptor structures within 30 kcal/mol using Glide XP (extra precision) (GLIDE, version 5.7, Schrödinger, LLC, New York, NY, 2011). The most favorable binding conformations of each receptor and ligand complex were selected.

### Ensemble Docking with Virtual Screening Workflow (VSW)

Ensemble docking using the Virtual Screening Workflow in Maestro 9.2 [35] was performed against the multiple fixed receptor conformations generated by IFD. The grids of receptor conformations selected from IFD were centered on the bound ligands with default box sizes. The Glide XP docking of prepared ligands was carried out using flexible docking with the OPLS 2005 force field. The regular XP docking with the prepared receptors was also conducted with the same grids and parameters used in the ensemble docking. The best docked poses with the lowest Glide score were selected for comparison.

## Results and Discussion

Recent studies reported the key protein-ligand interactions for known DNMT inhibitors using a number of molecular modeling techniques. However, most of the docking studies published so far have been conducted using only the catalytic domain of DNMT1 with a rigid structure of the protein. For several inhibitors, the actual binding site is unknown. Herein, we conducted IFD of novel inhibitors having “long” scaffolds, SGI-1027 and CBC12, considering receptor flexibility of DNMT1 and DNMT3A. For DNMT1, the whole structure (that consists of N-terminal and C-terminal domain), and only the catalytic domain were used during IFD. The different binding sites of DNMT1 and DNMT3A were explored for SGI-1027 and CBC12.

### Crystal Structures of DNMT1

**Figure 4:**
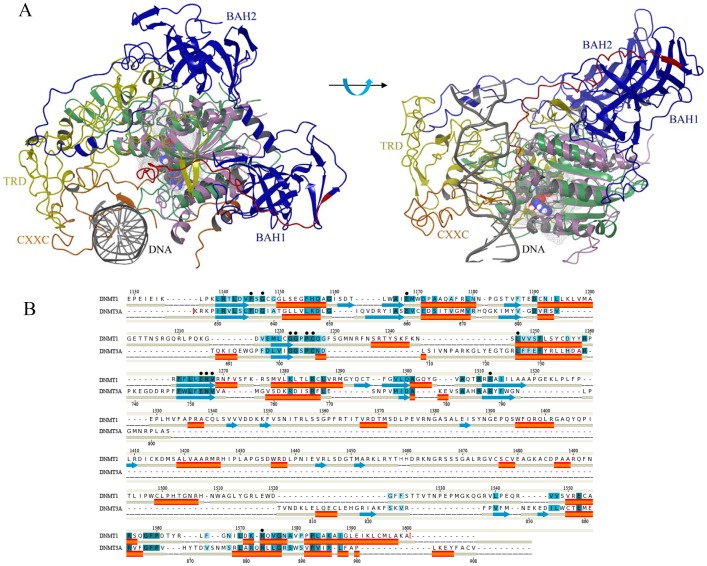
Comparison of the structures of DNMT1 and DNMT3A. (A) Structure alignment of MTase with other domains of DNMT1 and DNMT3A. The BAH1, BAH2, CXXC, autoinhibitory linker, TRD region and MTase domain of DNMT1 are colored in blue, orange, red, yellow and pink, respectively. The MTase domain of DNMT3A is colored in green and bound SAH is in space fill representation. (B) Sequence alignment of MTase domain of DNMT1 and DNMT3A. Weak-to-identical sequence similarities are colored in hues graded from light blue to dark blue. Identical residues interacting with ligands have been indicated with dots. Red cylinder and blue arrows represent helices and β-strands, respectively.

Two crystallographic structures of hDNMT1 were recently published. The structure bound with SAH and DNA containing unmethylated CpG sites was revealed first with a resolution of 3.6 Å (PDB id: 3PTA) [32]. Recently, hDNMT1 in complex with SFG was published with a resolution of 2.49 Å (PDB id: 3SWR). The two crystal structures are similar with RMSD of 1.4 Å (Figure 4A), hence the later crystal structure, with the lower resolution, was used in this study. Both structures are composed of N-terminal domain, including tandem bromo-adjacent homology (BAH1/2) and CXXC, and C-terminal methyltransferase (MTase) domain (Figure 1). A loop extended from BAH2 interacts with the target recognition domain (TRD) of the MTase domain. CXXC and BAH1 are connected with an auto inhibitory linker between DNA and the active site of DNMT1 (Figure 4A). According to the recently identified auto inhibitory mechanism [36], the CXXC domain interacts with DNA and drives the autoinhibitory linker to a position that prevents interaction between unmethylated DNA and the active site of MTase domain. In contrast, in the presence of hemimethylated DNA, the autoinhibitory linker does not block the active site, and the target DNA can be positioned at the substrate binding site resulting in the CpG methylation.

### Comparison of the Structures of DNMT1 and DNMT3A

Figure 4B shows the sequence alignment of the C-terminal domain of DNMT1 and DNMT3A. Although the size of the target recognition domains (TRD) between motifs VIII and IX of DNMT1 and DNMT3A are different, the C-terminal domain of DNMT3A superimposes well with the MTase domain of DNMT1 (Figure 4A). The key amino acid residues for the catalysis and cofactor binding are conserved [9]: (i) ENV motif (motif IV: Glu1266/752, Asn1267/753, Val1268/754) and RXR motif (motif VIII: Arg1310/786, Arg1312/788) (ii) F1145/636 and E1168/660 of the equivalent residue numbers in DNMT1 and DNMT3A (Figure 4B).

### Validation of the Docking Method

Before docking SGI-1027 and CBC12, we tested the Glide XP docking protocol to evaluate its capability to reproduce the binding mode of the co-crystallized SAH and SFG. SAH and SFG bound to the crystal structures of DNMT3A and DNMT1 were used as references to re-dock them into their corresponding binding sites. The RMSD values between the crystallographic and predicted conformations of SAH and SFG were 0.81 Å and 0.72 Å, respectively. These results showed the capability of the docking protocol to reproduce the binding mode of SAH and SFG (Figure S1).

### Binding Modes of SGI-1027 and CBC12 in the MTase Domain of DNMT3A

**Figure 5:**
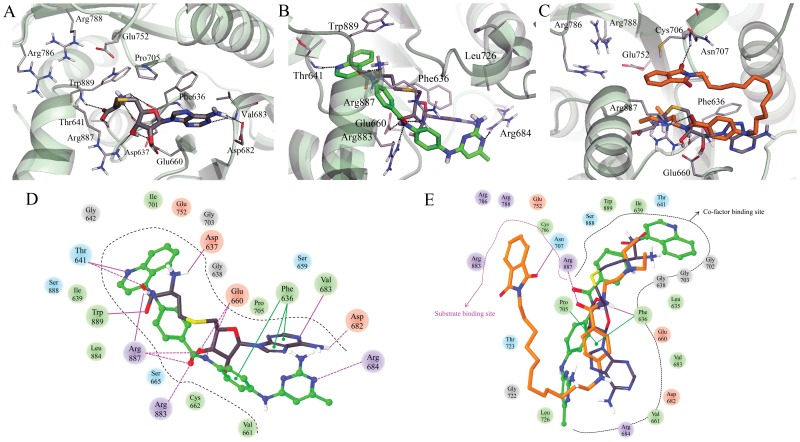
Induced-fit docking results of (A) SAH (carbon atoms in black), (B) SGI-1027 (carbon atoms in green), and (C) CBC12 (carbon atoms in orange) in the MTase domain of DNMT3A. Comparison of the interaction diagram (D) between SAH and SGI-1027, and (E) CBC12. Acidic, hydrophobic, basic, polar, and other residues at the active site are represented by red, green, purple, blue, and gray spheres, respectively. Hydrogen bonds between the ligand and backbone or side chains are shown in solid or dashed pink lines.

IFD was carried out to investigate the interaction between DNMT3A and the novel ligands. A total of 11 and 9 poses showing similar docked conformations of SGI-1027 and CBC12 were produced, respectively. The major forms of SGI-1027 and CBC12 were selected for comparison; the summary of the IFD results for each ligand is shown in Table 1. The top scored pose of SGI-1027 did not change significantly with a RMSD of 1.14 Å relative to the initial structure of 2QRV in complex with SAH. The residues Cys662, Gly703, Leu726, Arg883, and Trp889, within a distance of 4 Å from the docked SGI-1027, moved considerably from their starting position (RMSD>1 Å). Figure 5 shows the top scored binding poses and schematic 2D representation of SGI-1027 and CBC12 compared to the reference SAH (Figure 5A). SGI-1027 occupies the binding site of the cofactor, SAH (Figure 5B and D). The quinolylamino benzamide group of SGI-1027 forms hydrogen bonds with the backbone of Thr641 and the side chain of Arg883, Arg887, and Glu660. Of note, the L-homocysteine and two oxygen atoms of the ribose ring of SAH also make a hydrogen bond with the side chains of Thr641 as well as Glu660, which is a conserved residue in motif II of the methyltransferases (Figure 5A) [37]. The benzyl aminopyrimidine group of SGI-1027 occupies a region similar to the aminopurine ring of SAH and forms a hydrogen bond with the side chain of Arg684. This residue is located in the helix of DNMT3A-3L interface, and it is involved in the hydrogen bonding network between DNMT3A and DNMT3L in the crystal structure (PDB id: 2QRV) [4]. In addition, a benzene ring of both SGI-1027 and aminopurine ring of SAH makes π-π stacking interactions with Phe636, which is located in motif I.

**Table 1:** Summary of induced-fit docking results of SGI-1027 and CBC12 into the MTase domain of DNMT1 and DNMT3A with/without other domains.

Isoform (PDB)	Ligand	Cα RMSD (Å)^a^	Residues within 4 Å (RMSD)^b^
DNMT3A (2QRV)	SAH	1.10	F636, D637, G638, I639, T641, S659, E660, V661, C662, G681, D682, V683, R684, G703, S704, P705, L726, R887, S888, W889
	SGI-1027	1.14	S634, F636, D637, G638, I639, A640, T641, G642, Y656, E660, V661, C662 (1.91), S665, D682, R684, I701, G703 (1.71), P705, L726 (1.04), F866, R883 (1.15), L884, R887, S888, W889 (1.48)
	CBC12	0.23	L635, F636, D637, G638, I639, T641, S659, E660, V661, D682, V683, R684, G702, G703, P705, C706, N707, G722 (1.13), T723 (1.00), L726, E752, R883, G886, R887, S888, W889
DNMT1 OnlyMTase domainof 3SWR	SFG	0.08	F1145, S1146, G1147, C1148, G1149, G1150, L1151, I1167, E1168, M1169, W1170, A1173, E1189, D1190, C1191, N1192, G1223, P1225, L1247, E1266, N1578, A1579, V1580
	SAH	0.20	D1143, F1145, S1146, G1147, C1148, G1149, G1150, L1151, I1167, E1168, M1169, W1170, A1173, E1189, D1190, C1191, N1192, G1222, G1223, P1225, L1247, E1266, R1312, N1578, A1579, V1580
	SGI-1027	0.22	V1144, F1145, S1146, G1147, I1167, E1168, M1169, W1170, E1189, D1190, C1191, G1223, P1225, L1247, E1266, V1268, R1310, R1312, T1525, T1526, V1527, T1528, Q1536, G1577, N1578, A1579, V1580
	CBC12	0.22	D1143, V1144, F1145, S1146, G1147, G1149, G1150, L1151, C1221, G1222, G1223, P1225, C1226, Q1227, G1228, S1230, L1264, E1266, N1267, V1268, R1269, F1274, R1310, R1311, R1312, T1525, T1528, Q1536, G1577, N1578, A1579, V1580
DNMT1 Wholestructure of3SWR	SGI-1027	0.10	R650, M694, A695, M696, K697 (1.13), E698, A699 (1.10), D1143, V1144, F1145, S1146, G1147, C1148, G1149, G1150, L1151, S1152, E1168, M1169, W1170, P1172, F1177, C1221, G1222, G1223, P1225, Q1227, F1559, D1571, R1574, Q1575, N1578, A1579, V1580
	CBC12	0.11	R650, M694, A695, M696, K697, E698 (1.31), A699 (1.51), D700, D701, D1143, V1144, F1145, S1146, G1147, G1149, G1150, L1151, S1152, M1169, W1170, C1221, G1222 (1.06), G1223, P1224, P1225, C1226, Q1227, L1264, E1266, N1267, V1268, R1312, L1570,D1571, R1574, N1578, A1579, V1580

a The average RMSD of the Cα atoms of superimposed proteins between IFD structure and initial structure.

b Residues within a distance of 4 Å from the docked inhibitor. Residues participating in interaction with docked inhibitor are underlined. The conformational changes of residues with RMSD ≥1 Å are shown in brackets.

**Figure 6:**
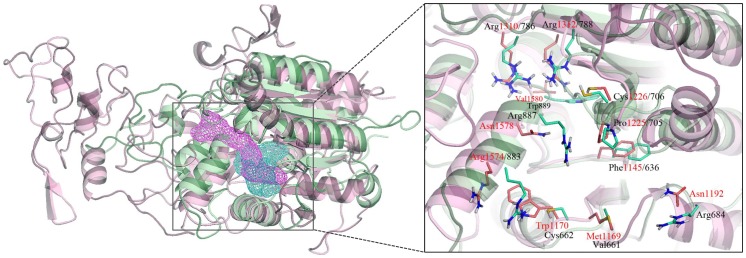
Structure alignment of MTase domain of DNMT1 (pink ribbon) and DNMT3A (green ribbon) after induced-fit docking. The binding sites of SGI-1027 in DNMT1 and DNMT3A are represented by pink and green mesh, respectively. Comparison of the side chain conformations between DNNT1 and DNMT3A in the substrate and cofactor binding sites is shown in the enlarged box. The amino acid residues of DNMT1 (carbon atoms in pink) and DNMT3A (carbon atoms in green) are indicated with a red and black number, respectively.

The structure of the top scored binding pose of CBC12 is almost the same (RMSD of 0.22 Å) as the initial structure of 2QRV (Figure 5C and E). Only two residues of Gly722 and Thr723 within a distance of 4 Å from the docked CBC12, had a RMSD>1 Å. CBC12, which has a longer scaffold than SGI-1027, occupies the cofactor and substrate binding sites of DNMT3A. The procainamide moiety of CBC12 is docked into the cofactor site in a similar manner to SAH and SGI-1027. The amide group forms hydrogen bonds with the backbone of Phe636 and the side chain of Arg887, which are observed in an IFD pose of SGI-1027. In addition, the benzene ring makes π-π stacking interactions with Phe636 and makes contacts with Pro705, which are located in motif IV of the substrate binding site. In contrast, the phthalimide moiety of CBC12 is positioned close to the substrate binding site forming a hydrogen bond with the backbone of Asn707 next to the catalytic cysteine residue. Although this binding mode of CBC12 is different from the recently published docking result [30], it is quite reasonable for the comparison of DNMT1 and DNMT3A structures (Figure 6). Two residues of DNMT3A, namely; Arg887 (corresponding to Asn1578 in DNMT1) and conserved Pro705 are blocking the aisle between the cofactor and substrate binding sites. Therefore, CBC12 occupies the cofactor binding site, close to the substrate binding site, forming a U-shape. This result is different from the binding mode of SGI-1027 into the MTase domain of DNMT1 without other domains (see below).

### Docking of SGI-1027 and CBC12 in the MTase Domain of DNMT1 in the Absence of other Domains

**Figure 7:**
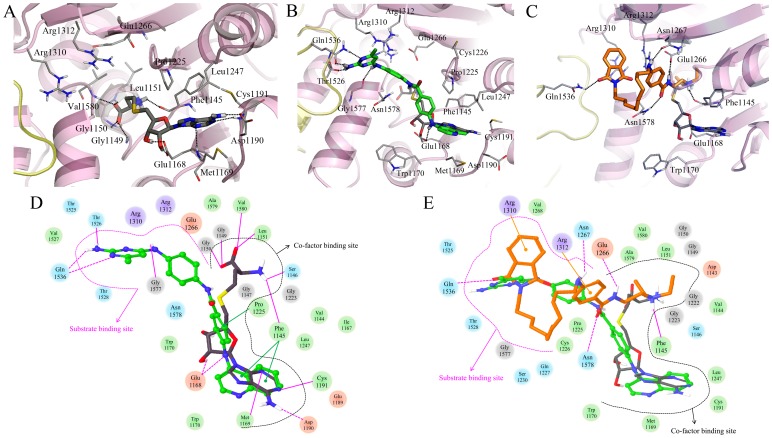
Induced-fit docking results of (A) SAH (carbon atoms in black), (B) SGI-1027 (carbon atoms in green), and (C) CBC12 (carbon atoms in orange) with the MTase domain of DNMT1. TRD region is represented by yellow loop. Comparison of the interaction diagram (D) between SAH and SGI-1027, and (E) CBC12. Acidic, hydrophobic, basic, polar, and other residues at the active site are represented by red, green, purple, blue, and gray spheres, respectively. Hydrogen bonds between the ligand and backbone or side chains are shown in solid or dashed pink lines.

The MTase domain of hDNMT1 without other domains was used for the IFD of SGI-1027 and CBC12 using SAH as a reference. A total of 15 poses for SGI-1027, and 9 poses for CBC12 were obtained and the preferred binding mode for each compound was selected for further analysis. The selected structures had small changes (RMSD <0.3 Å) compared to the initial structure. Residues within a distance of 4 Å from the docked inhibitor showed a RMSD <1 Å relative to their starting position. The binding pose of SAH was superimposed with a RMSD of 1.1 Å on the crystal ligand, SFG (Figure 7A). A summary of the IFD results is shown in Table 1.

The best docked conformation of SGI-1027 occupies the cofactor and substrate binding sites (Figure 7B). The 2D-interaction diagram clearly shows the different binding modes of SGI-1027 between DNMT1 and DNMT3A (Figures 7D and 5D). The quinoline amine group of SGI-1027 was docked in the cofactor binding site similar to the aminopurine ring of SAH, and forms a hydrogen bond with Glu1168 corresponding to Glu660 in DNMT3A. Both of quinoline and aminopurine rings make π-π stacking interactions with Phe1145 that are related interactions observed with the equivalent Phe636 in DNMT3A. The benzene ring of quinolylamino benzamide group is positioned between the cofactor and substrate binding sites making contacts with the conserved Pro1225 corresponding to Pro705 in DNMT3A. The benzyl amino pyrimidine group of SGI-1027 was docked in the substrate binding site, in the ENV and RXR motifs. The amino pyrimidine moiety forms hydrogen bonds with the backbone of Gly1577 and Thr1526 as well as the side chain of Gln1536 in the TRD region. Of note, this moiety is located in the site that can cause bumping into Asp700–702 in autoinhibitory linker in case it was present. Actually, the autoinhibitory domain located close to the substrate binding site, and negatively charged residues such as Glu703 and Glu698 in this place make a hydrogen bond with Gln1536, Arg1574, and Asn1578, respectively.

CBC12 was docked in the cofactor and substrate binding sites (Figure 7C and E). The diethyl amino group of the procainamide moiety of CBC12 occupied a region similar to the L-homocysteine of SAH, and the positively charged amino group forms a hydrogen bond with backbone of Phe1145. The amino benzamide group of procainamide and phthalimide moieties occupied the substrate binding site similar to the benzyl amino pyrimidine group of SGI-1027 as shown in Figure 7. The amino benzamide group forms a hydrogen bond with the side chains of Asn1267 and Glu1266 in the ENV motif, and Asn1578. In addition, a π-cation interaction was also observed between the benzene ring and Arg1312 that participate in the mechanism of methylation [26]. The phthalimide moiety forms a hydrogen bond with the side chain of Gln1536 in the TRD region, similar to the amino pyrimidine moiety of SGI-1027, and makes π-cation interactions with Arg1310 in the RXR motif.

The IFD results obtained considering only the MTase domain of DNMT1 suggest that the binding of SGI-1027 or CBC12 blocks the interaction between DNA and the substrate binding site.

### Docking of SGI-1027 and CBC12 in the MTase Domain of DNMT1 in the Presence of other Domains

**Figure 8:**
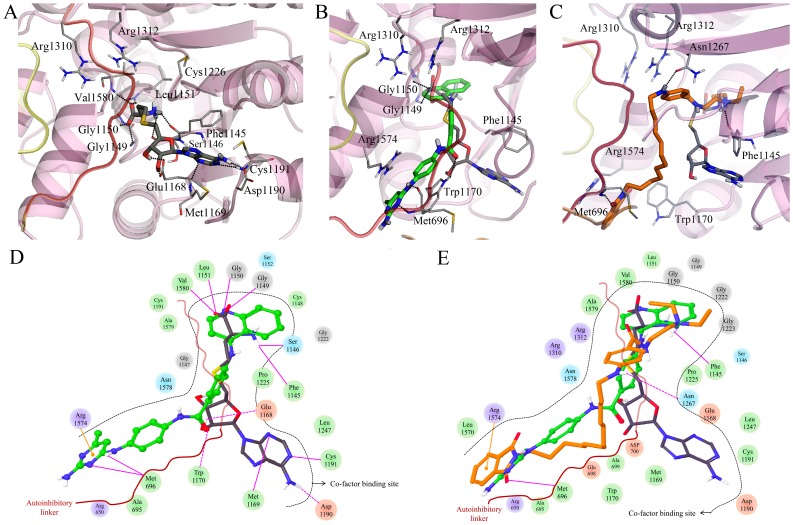
Induced-fit docking results of (A) SAH (carbon atoms in black), (B) SGI-1027 (carbon atoms in green), and (C) CBC12 (carbon atoms in orange) in the MTase domain of DNMT1 in the presence of other domains. TRD region and autoinhibitory linker are represented by yellow and red loop, respectively. Comparison of the interaction diagram (D) between SAH and SGI-1027, and (E) CBC12. Acidic, hydrophobic, basic, polar, and other residues at the active site are represented by red, green, purple, blue, and gray spheres, respectively. Hydrogen bonds between the ligand and backbone or side chains are shown in solid or dashed pink lines. The π-cation interactions are indicated with orange lines.

The structure of full-length DNMT1 composed of the N-terminal including other domains, and the C-terminal catalytic methyltransferase domain was recently published. The autoinhibitory mechanism was identified from this structure concluding that the CXXC domain and autoinhibitory linker play an important role in this mechanism [32]. Therefore, we considered the docking studies into the MTase domain of DNMT1 in the presence of other domains. A total of 15 poses for SGI-1027, and 6 poses for CBC12 were obtained by IFD. The binding mode of SAH used as a reference was identical to that with only C-terminal catalytic domain of DNMT1 (Figure 8A and 7A). Each of the top scored IFD pose in complex with SGI-1027 and CBC12 had few changes from the initial structure of 3SWR (RMSD <1 Å). Table 1 summarizes the IFD results for each ligand. Only two (i.e., Lys697 and Ala699) and three residues (i.e., Glu698, Ala699, and Gly1222) within a distance of 4 Å from the docked SGI-1027 and CBC12 slightly moved (RMSD>1 Å) from their starting positions, respectively. In contrast, the selected top binding mode of SGI-1027 and CBC12 are substantially different from the IFD results into the only C-terminal catalytic domain of DNMT1.

SGI-1027 was docked into the cofactor binding site making contacts with the autoinhibitory linker (Figure 8B and D). The quinolylamino benzamide group of SGI-1027 occupies a region similar to the L-homocysteine of SAH. The quinoline ring forms hydrogen bonds with the backbone of Gly1149 and Gly1150; the same interactions are observed for SAH. A hydrogen bond interaction between the amide moiety of quinolylamino benzamide group and the side chain of Trp1170 is also found. The benzyl amino pyrimidine group of SGI-1027 stretches parallel to the autoinhibitory linker in the opposite direction of the aminopurine ring of SAH. The amino pyrimidine ring forms a hydrogen bond interaction with the backbone of Met696 in the autoinhibitory linker. The same ring also makes a π-cation interaction with Arg1574 in motif X, which is a conserved residue in DNMT3A. Of note, these interactions with the autoinhibitory linker are not found for SAH.

Interestingly, the binding modes of CBC12 and SGI-1027, both compounds with “long” scaffolds, are similar (see Figure 8B, 8C, and 8E). The diethyl amino group of the procainamide moiety of CBC12 occupies a region similar to the quinolylamino group of SGI-1027 and the L-homocysteine of SAH. The positively charged amino group forms a hydrogen bond with the backbone of Phe1145. This interaction is also found between the positively charged amino group of SAH and the backbone of Phe1145 (Figure 8A and 8D). The amino benzamide group of the procainamide moiety occupies the substrate binding site and forms a hydrogen bond with side chain of Asn1267 in the ENV motif. The phthalimide moiety with alkyl linker was docked parallel to the autoinhibitory linker with the similar binding mode to the benzyl amino pyrimidine group of SGI-1027. The phthalimide forms a hydrogen bond with the backbone of Met696 and makes π-cation interactions with Arg1574.

The IFD results with whole structure of DNMT1 suggest that the binding of SGI-1027 or CBC12 in the presence of unmethylated DNA helps to stabilize the position of the autoinhibitory linker between DNA and the substrate binding site of MTase domain by additional interactions with residues in the autoinhibitory linker as well as with the cofactor binding site.

### Comparison of the IFD, Ensemble Docking, and Regular XP Docking

We compared the binding scores obtained with different docking methods and the reported activity of SAH, SGI-1027, and CBC12. Table 2 summarizes the docking scores. The IFD results are remarkable in that the XP scores of SGI-1027 docked to the DNMT1 and DNMT3A are more favorable than the corresponding scores of SAH. This is in excellent agreement with the *in vitro* data recently published showing that SGI-1027 inhibits the activity of DNMT directly by competing with the cofactor [27]. Furthermore, there is a very good agreement between the similar binding energies of SGI-1027 with DNMT1 and DNMT3A and the inhibitory activity of this compound towards both isoforms. Datta J. et al. indicates that SGI-1027 is the non-selective inhibitor to the DNMT1 and DNMT3A [27]. Therefore, the docking results of SGI-1027 and SAH have a remarkable agreement with this experimental result. CMB12 shows comparable binding energies with SGI-1027. This is in accord with the biological activity reported for CBC12 that showed better activity than the inhibitors procainamide and RG108 [30].

**Table 2:** XP scores of regular docking, induced-fit docking and ensemble docking of SGI-1027 and CBC12 into the MTase domain of DNMT1 and DNMT3A with/without other domains.

Regular XP docking score (kcal/mol), (RMSD)^a^			
Ligand	hDNMT3A	hDNMT1	
	MTase domain	MTase domain	MTase domain with N-terminal domain
SAH	−8.8	−8.0	−8.7
SGI-1027	−5.8 (4.5)	−2.7 (4.6)	−5.8 (8.6)
CBC12	−4.6 (8.3)	−4.5 (6.2)	−4.7 (9.9)
**Induced-fit docking score (kcal/mol)**			
**Ligand**	**hDNMT3A**	**hDNMT1**	
	**MTase domain**	**MTase domain**	**MTase domain with N-terminal domain**
SAH	−**8.8**	−**7.8**	−**10.9**
SGI-1027	−**9.5**	−**9.2**	−**11.6**
CBC12	−**7.4**	−**8.7**	−**10.3**
**Ensemble docking score (kcal/mol)**			
**Ligand**	**hDNMT3A**	**hDNMT1**	
	**MTase domain**	**MTase domain**	**MTase domain with N-terminal domain**
SAH	−9.3	−7.7	−10.2
SGI-1027	−10.4	−7.5	−11.1
CBC12	−7.7	−7.8	−8.1

a RMSD ≥1 Å of ligand compared with binding mode from IFD are shown in brackets.

In addition, the ensemble docking with top selected IFD poses of each ligand was performed. Although the binding poses of ligands using multiple receptor conformation are very similar to the IFD poses (RMSD <1 Å), the ensemble docking energies of SGI-1027 considering only the MTase domain and CBC12 in the whole structure of DNMT1, slightly increased compared to the IFD energies. To investigate the effect of IFD, we also conducted regular XP docking of SAH, SGI-1027, and CBC12 with the rigid structure of DNMT1 and DNMT3A. Regular XP docking was performed with the same methods implemented in ensemble docking. Interestingly, some parts of ligands were docked in different pockets that do not correspond to the binding site obtained with IFD (Figure S2). For example, the benzyl amino pyrimidine group of SGI-1027 did not occupy the substrate binding site in the docking with only the MTase domain of DNMT1. In the whole structure of DNMT1, the quinolylamino benzamide group of SGI-1027 was docked outside the cofactor binding site similar to the aminopurine ring of SAH. Furthermore, the interaction of SGI-1027 with Arg684 in DNMT3A is not feasible in the regular docking. Their binding poses changed substantially (RMSD>4 Å) from the top ranked poses obtained with IFD (Table 2). The conformational changes of the ligands at the binding site resulted in a dramatic increase of the binding energies.

Taken together, the findings discussed above suggest that IFD provides reasonable binding pose and scores for the novel ligands taking into account possible movements of several side chains.

### Proposed Inhibitory Mechanism of SGI-1027 of DNMT1

The major differences in the docking results discussed above are the proposed binding modes of SGI-1027 and CBC12 in the MTase domain with or without other domains. Indeed, in the whole crystal structure of DNMT1 corresponding to the unmethylated state, the autoinhibitory linker is positioned between the DNA and the active site preventing the entrance of DNA into the substrate binding site. In contrast, the autoinhibitory linker is outside the active site in the hemimethylated state corresponding to the MTase domain only. Interestingly, the binding conformation of SGI-1027 and CBC12 in the MTase domain occupies the cofactor and substrate binding sites. Conversely, in the whole structure of DNMT1, SGI-1027 and CBC12 were docked into the cofactor binding site, similar to the conformation of the co-crystallized SAH, and both compounds interact with amino acid residues of the autoinhibitory linker.

**Figure 9:**
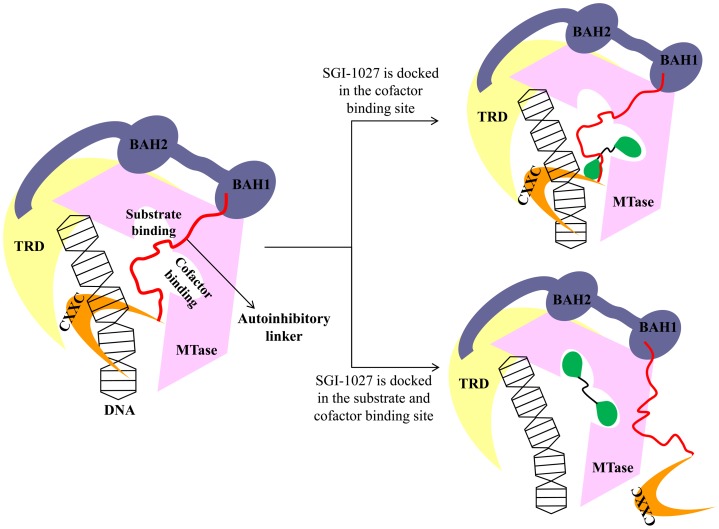
Proposed inhibitory mechanism of SGI-1027 in DNMT1.

Based on these results, we proposed two possible inhibition mechanisms by ligand docking with hDNMT1 in the unmethylated or hemimethylated state (Figure 9). In the presence of the MTase with other domains corresponding to unmethylated state, SGI-1027 or CBC12 is tightly bound to the autoinhibitory linker as well as to the cofactor binding site. Consequently, the autoinhibitory linker is stabilized between the active site of the MTase domain and DNA which results in preventing access of target DNA to the substrate binding pocket. In contrast, SGI-1027 or CBC12 is docked in the cofactor and substrate binding sites in the presence of only MTase domain corresponding to the hemimethylated state. The docking results suggest that the bound inhibitors may act as an autoinhibitory linker in the substrate binding site and also block the cofactor binding site. A second hypothesis is that the autoinhibitory linker cannot enter the active site due to the presence of the inhibitor, and it is pushed out of the cleft formed by the catalytic core and the TRD domain. Indeed, steric clashes are predicted between bound SGI-1027 or CBC12 and the autoinhibitory linker at the substrate binding site when they are superimposed on the whole structure. The putative interaction of SGI-1027 and CBC12 with the enzymes can potentially be verified using saturation transfer difference NMR spectroscopy experiments as recently reported for L-RG108 and phthalimide [30].

It is remarkable that SGI-1027 and CBC12 showed similar binding modes. Both compounds support the notion that “long” scaffolds seem to be beneficial for the generation of novel inhibitors. In addition, two proposed mechanisms using our approaches are applicable to other unknown inhibitors. The binding modes of other inhibitors in the presence of the autoinhibitory loop have a potential to be changed because the autoinhibitory loop is located closed to the active site. Therefore, the novel hypothesis can provide new approaches and insights for the design and discovery of new inhibitors of DNMT.

### Conclusions

The goal of this study was to explore the binding site and to propose docking models for SGI-1027 and CBC12, which are novel DNMT inhibitors with “long” scaffolds. To date, most of the docking studies of DNMT inhibitors with similar size have been performed at the substrate binding site of the MTase domain of DNMTs. In this study, we conducted IFD of ligands with the cofactor and substrate binding sites in the MTase domain of human DNMT1 and DNMT3A in the presence and absence of other domains. To the best of our knowledge, this is the first docking study in the MTase domain of human DNMTs in the presence of other domains. In the proposed binding model with DNMT3A, SGI-1027 occupies the cofactor binding site, and it has a similar binding mode as SAH whereas CBC12 is docked in the substrate binding site as well as the cofactor binding site. In DNMT1, the binding mode of SGI-1027 and CBC12 in the MTase domain depend on the presence of other domains. SGI-1027 and CBC12 occupy the cofactor and substrate binding sites when the docking was conducted in the MTase domain only. According to this model, the bound inhibitors work like the autoinhibitory linker and prevent the entrance of DNA into the substrate binding site. Docking with DNMT1 in the presence of other domains revealed that SGI-1027 and CBC12 may occupy the cofactor site, similar to SAH. Additional interactions with the autoinhibitory linker may help to maintain such linker in a position between the active site and DNA. These hypotheses are in agreement with the reported autoinhibitory mechanism [32], [36].

The binding score of SGI-1027 is more favorable than the corresponding score of SAH. This is in excellent agreement with the *in vitro* data. Furthermore, the similar binding energies of SGI-1027 with DNMT1 and DNMT3A indicate that SGI-1027 is a non-selective inhibitor as shown in the experimental result [27]. It is remarkable the related binding modes of CBC12 and SGI-1027. The docking result of CBC12 supports the proposed inhibitory mechanism and suggests that “long” scaffolds would be beneficial for the generation of novel DNMT inhibitors. These comprehensive analyses provide the insights for further design and development of new scaffolds for DNMT inhibitors. Indeed, the chemical structures of CBC12 and SGI-1027 are significantly longer than the structures of several small-molecule DNMT inhibitors. The outcome of this work suggests that it is expected that small-molecules with three or more rings, linked by the appropriate connectors, may either, mimic the function of the autoinhibitory linker or stabilize the position of the linker. Therefore, one of the next logical steps of this work is to test synthetic or commercial structural analogues of SGI-1027 and CBC12 considering the SAR already available for these lead compounds. A related following step is to perform a computational shape- and pharmacophore-based screening of existing or virtual compound libraries with the aim of identifying promising compounds with long and novel scaffolds for experimental validation.

## Supporting Information

Figure S1 **Validation of the docking protocol comparing the predicted binding modes of SAH and SFG with the co-crystallized ligands.**
(DOC)

Figure S2 **Comparison of the binding modes of SGI-1027 with induced-fit and regular XP docking.**
(DOC)
