# Supplementary material for: Molecular Modeling Studies of the Novel Inhibitors of DNA Methyltransferases SGI-1027 and CBC12: Implications for the Mechanism of Inhibition of DNMTs
Source: PLoS One. 2013 Apr 25;8(4):e62152. doi: 10.1371/journal.pone.0062152 (PMC3636198; doi:10.1371/journal.pone.0062152)
Supplement: Figure S1 — Validation of the docking protocol comparing the predicted binding modes of SAH and SFG with the co-crystallized ligands. (DOC) [file pone.0062152.s001.doc]

**
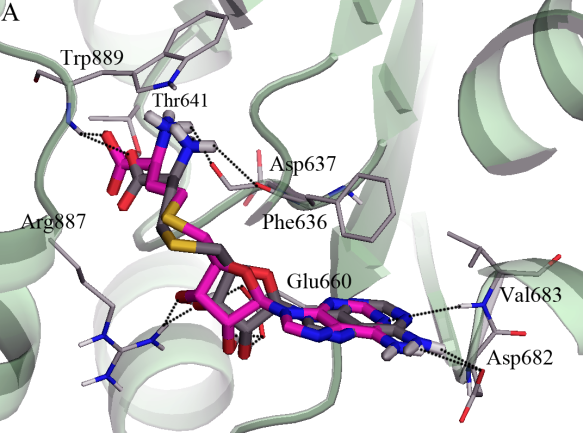

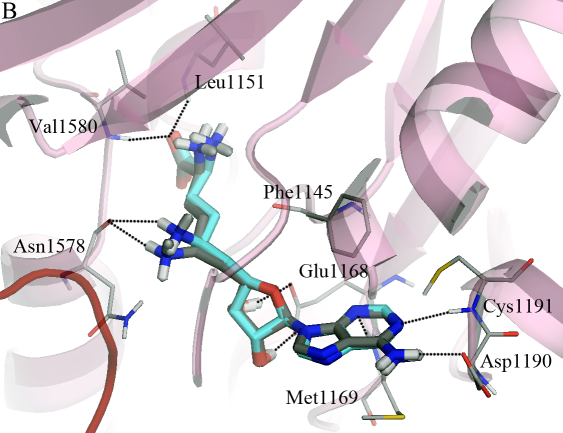
**

**Figure S1.** Validation of the docking protocol comparing the predicted binding modes of (A) SAH (carbon atoms in pink) in DNMT3B and (B) SFG (carbon atoms in sky blue) in DNMT1 with the co-crystallized ligands SAH and SFG (carbon atoms in black), respectively.
