# Supplementary material for: Molecular Modeling Studies of the Novel Inhibitors of DNA Methyltransferases SGI-1027 and CBC12: Implications for the Mechanism of Inhibition of DNMTs
Source: PLoS One. 2013 Apr 25;8(4):e62152. doi: 10.1371/journal.pone.0062152 (PMC3636198; doi:10.1371/journal.pone.0062152)
Supplement: Figure S2 — Comparison of the binding modes of SGI-1027 with induced-fit and regular XP docking. (DOC) [file pone.0062152.s002.doc]

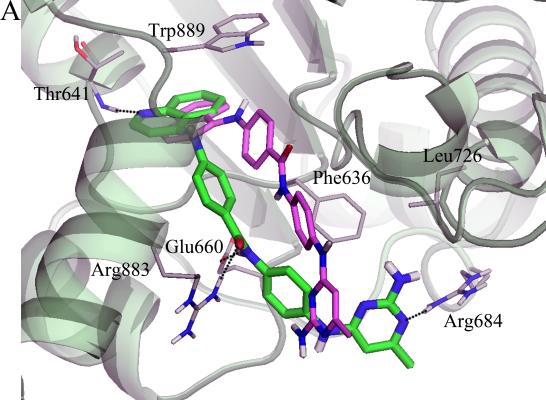

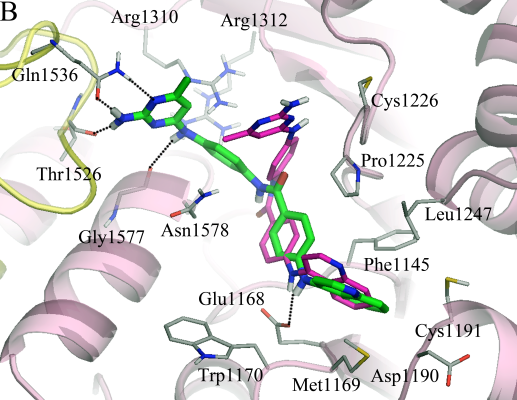

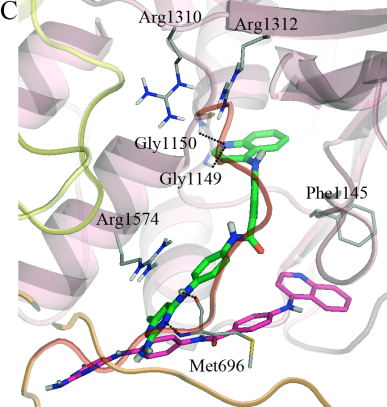


**Figure S2.** Comparison of the binding modes of SGI-1027 with induced-fit docking (carbon atoms in green) and regular XP docking (carbon atoms in pink) in the (A) DNMT3A, (B) MTase domain and (C) MTase with other domains of DNMT1.
